# Adjunctive ab-interno goniotomy in chronic angle-closure glaucoma: a retrospective proof-of-concept pilot study using doubly robust learning

**DOI:** 10.3389/fopht.2026.1846121

**Published:** 2026-06-19

**Authors:** YuQi Ren, Xiaojing Zha, YiZheng Zhang, Jin Xuan, ZhiYong Meng, ChenMing Zhang

**Affiliations:** 1 Department of Ophthalmology, Shaowu Municipal Hospital, Shaowu, Fujian, China; 2 Department of Ophthalmology, Shaowu City Traditional Chinese Medicine Hospital, Shaowu, Fujian, China; 3 Department of Oncology, Shengli Clinical Medical College of Fujian Medical University, Fujian Provincial Hospital, Fuzhou University Affiliated Provincial Hospital, Fuzhou, China

**Keywords:** ab-interno goniotomy, anterior segment optical coherence tomography, causal inference, chronic angle-closure glaucoma, conditional average treatment effects, doubly robust learning, goniosynechialysis, phacoemulsification

## Abstract

**Purpose:**

To assess whether an anterior segment optical coherence tomography (AS-OCT)-informed causal framework could estimate the added benefit of adjunctive ab-interno goniotomy during phacoemulsification with goniosynechialysis (Phaco-GSL) in chronic angle-closure glaucoma (CACG).

**Design:**

Retrospective single-center comparative cohort pilot study.

**Methods:**

In a single-center development cohort of 102 eyes, 54 underwent phacoemulsification with goniosynechialysis alone and 48 underwent adjunctive ab-interno goniotomy. Marginal treatment effects were estimated using stabilized inverse probability of treatment weighting (IPTW) and augmented inverse probability weighting (AIPW). Medication-free complete success was defined as intraocular pressure (IOP) 5–18 mmHg with at least 20% reduction from baseline without IOP-lowering medications or additional glaucoma surgery; qualified success used the same IOP criteria with or without medications. Qualified failure was defined as loss of qualified success or need for additional glaucoma surgery. Time to qualified failure through 24 months was analyzed using stabilized IPTW-weighted Kaplan-Meier curves and weighted Cox models. Predicted individualized benefit estimates for 24-month medication-free complete success were derived from baseline clinical and AS-OCT features using a doubly robust learner with ridge regularization and 5-fold cross-fitting.

**Results:**

Adjunctive goniotomy was associated with higher 24-month qualified-failure-free survival under stabilized IPTW adjustment (0.682 vs 0.425; weighted hazard ratio 0.475, 95% CI 0.257–0.880; p=0.018). Doubly robust estimates favored adjunctive goniotomy for 24-month medication-free complete success (risk difference 0.291; 95% CI 0.105–0.478) and qualified success (risk difference 0.233; 95% CI 0.064–0.403). Adjusted mean IOP differences were modest, whereas medication-free complete success and medication burden showed more apparent separation. Hyphema occurred numerically more often with adjunctive goniotomy. Predicted individualized benefit estimates (model-predicted absolute probability differences) were variable and were not interpreted as clinically actionable recommendations.

**Conclusions:**

Adjunctive goniotomy was associated with more favorable 24-month surgical control in this retrospective pilot cohort. Imaging-informed doubly robust learning may support future studies of treatment-effect heterogeneity, but the current predicted individualized benefit estimates require external validation before clinical use.

## Introduction

Chronic angle-closure glaucoma (CACG) remains a major cause of irreversible blindness worldwide and disproportionately affects Asian populations. In eyes with CACG and visually significant cataract, phacoemulsification with goniosynechialysis (Phaco-GSL) is widely performed to deepen the anterior chamber and mechanically reopen the iridocorneal angle. However, anatomical reopening does not consistently translate into durable intraocular pressure (IOP) control (1). With long-standing synechial closure, irreversible trabecular alterations, such as fusion of trabecular lamellae and loss of endothelial cellularity, may create a persistent functional outflow bottleneck even when the angle can be successfully reconstructed (2, 3).

Adjunctive ab-interno goniotomy is therefore mechanistically appealing (4). By incising or excising a segment of high-resistance trabecular tissue, goniotomy may augment conventional aqueous outflow and potentially improve the probability of sustained postoperative IOP control beyond Phaco-GSL alone (5). Yet, its real-world effectiveness remains difficult to infer from routine retrospective data. In clinical practice, surgeons tend to selectively add goniotomy in eyes perceived to be at higher risk of failure, introducing confounding by indication that can bias naïve effectiveness comparisons (3, 6–8). Conventional multivariable regression can adjust for measured covariates, but its conclusions may depend heavily on correct specification of the outcome model and may not directly address the treatment-selection process. Doubly robust methods combine treatment-assignment and outcome models and can provide more robust estimates under standard measured-confounding assumptions when at least one of these models is adequately specified (9–12).

In current practice, decisions to add angle surgery often rely on pragmatic markers such as peripheral anterior synechiae (PAS) extent, medication burden, baseline IOP, glaucoma severity, history of acute angle-closure attacks, lens-related anatomy, prior laser peripheral iridotomy, medication response, and intraoperative angle appearance. These markers are clinically intuitive but imperfect, because they do not directly measure residual trabecular and distal outflow function. Consequently, some eyes may receive additional angle surgery despite being likely to succeed with Phaco-GSL alone, whereas others with occult functional outflow compromise may be undertreated (13–18).

Quantitative anterior-segment imaging provides reproducible anatomic phenotyping across the primary angle-closure disease spectrum. Anterior segment optical coherence tomography (AS-OCT) measures angle opening distance (AOD500/750), trabecular-iris space area (TISA500/750), trabecular-iris angle (TIA), anterior chamber depth (ACD), and lens vault (LV), while ultrasound biomicroscopy (UBM) can complement these measurements by characterizing ciliary body configuration and plateau iris. Integrating these baseline features with transparent causal methods may help identify anatomical and clinical patterns associated with larger or smaller incremental benefit of adjunctive goniotomy in future patient-selection studies (19–22).

Accordingly, we conducted a retrospective single-center proof-of-concept pilot study with two aims: (1) to estimate the average treatment effect of adjunctive ab-interno goniotomy on 24-month surgical outcomes using doubly robust methods under standard measured-confounding assumptions; and (2) to explore whether baseline clinical and imaging features could support hypothesis-generating assessment of treatment-effect heterogeneity. Internal stability checks and policy simulations were used only as methodological demonstrations and were not intended to generate clinically actionable treatment rules.

## Methods

### Study design, ethical oversight, and reporting standards

This retrospective comparative cohort study utilized data from a tertiary eye center in Fujian Province, China, and included consecutive patients treated between February 2021 and November 2023. This single-center cohort served as the development cohort for all causal and treatment-effect heterogeneity analyses.

**Figure 1:**
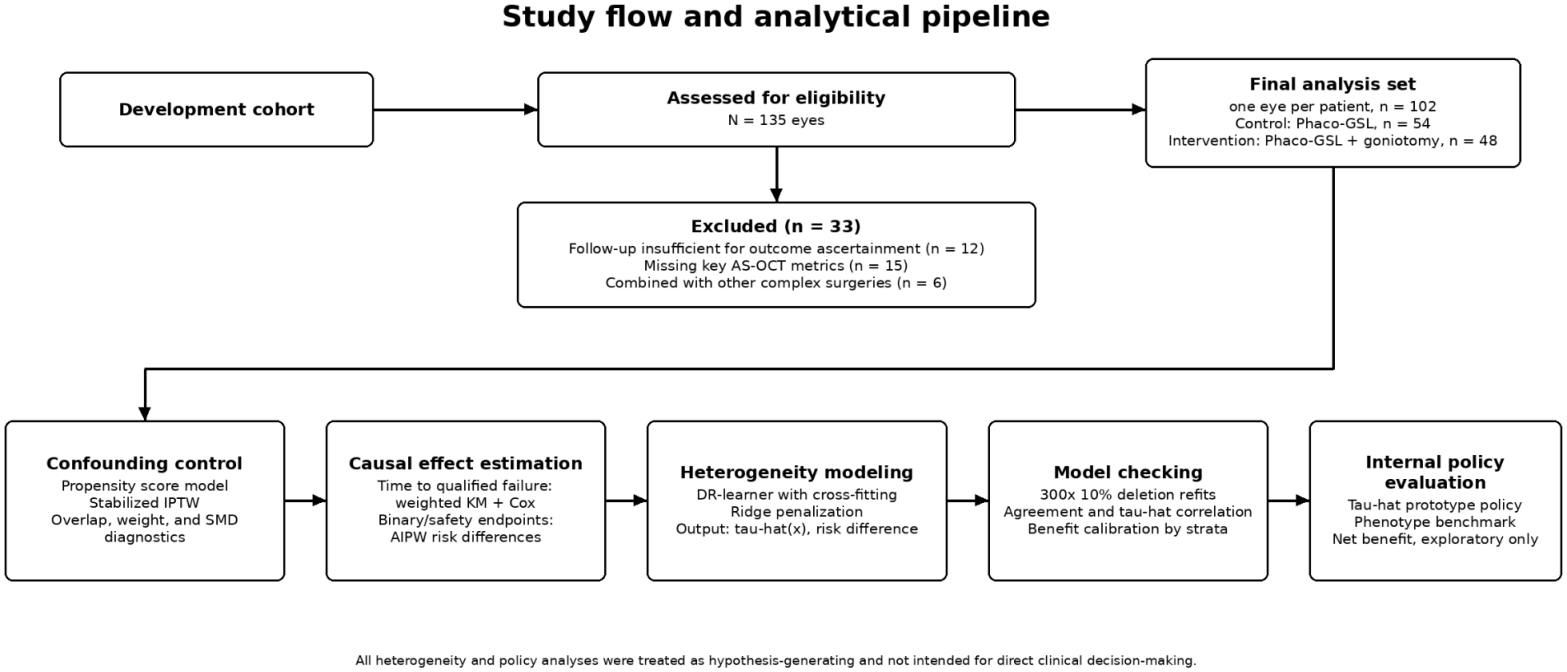
Study flow and analytical pipeline. The development cohort comprised eyes with chronic angle-closure glaucoma undergoing phacoemulsification with goniosynechialysis (Phaco-GSL) with or without adjunctive ab-interno goniotomy. After eligibility assessment and prespecified exclusions, 102 eyes (one eye per patient) were included (control: Phaco-GSL, n=54; intervention: Phaco-GSL + goniotomy, n=48). For the primary time-to-event endpoint (time to qualified failure, analyzed over 24 months), events and censoring were summarized by treatment group (control: 33 events, 1 censored before 24 months, 20 administratively censored at 24 months; intervention: 15 events, 0 censored before 24 months, 33 administratively censored at 24 months). Confounding by indication was addressed using a propensity score model and stabilized inverse probability of treatment weighting (IPTW), with overlap/weight diagnostics and post-weighting balance assessment using standardized mean differences (SMDs). Marginal causal effects were estimated using stabilized IPTW Kaplan-Meier curves and a weighted Cox model for time to qualified failure, and doubly robust augmented inverse probability weighting (AIPW) for binary endpoints and safety outcomes. Exploratory conditional average treatment effects (CATEs), summarized as predicted individualized benefit estimates (tau-hat), were estimated using a doubly robust learner (DR-learner) with cross-fitting and ridge penalization on the risk-difference scale (intervention - control). Model stability was assessed via repeated 10% random-deletion refits, and prototype policy evaluation was performed within the development cohort. Findings are hypothesis-generating and not intended for direct clinical decision-making.

The study adhered to the tenets of the Declaration of Helsinki, received approval from the Ethics Committee of Shaowu Municipal Hospital (approval no. 2023-4), and obtained written informed consent from all participants for surgical intervention and use of clinical data. The analytical objectives were causal and exploratory: (1) to estimate the average treatment effect (ATE) of adjunctive ab-interno goniotomy added to phacoemulsification with goniosynechialysis (Phaco-GSL), and (2) to explore conditional average treatment effects (CATEs), summarized as predicted individualized benefit estimates (tau-hat), and evaluate, in simulation, how tau-hat might inform prototype treatment strategies with internal stability checks. Reporting followed STROBE and was aligned with key items from TRIPOD+AI and PROBAST+AI, including transparent model specification, internal validation strategy, and control of bias/overfitting (23–25). Study flow and the analytical pipeline are summarized in **Figure 1**, with an additional stepwise workflow provided in **Supplementary Table 6**.

### Study population

Eligible participants were adults aged 50–80 years with coexisting chronic angle-closure glaucoma (CACG) and visually significant cataract. Inclusion criteria required medically uncontrolled intraocular pressure (IOP) >21 mmHg or controlled IOP requiring ≥2 topical glaucoma medications. Exclusion criteria included secondary angle-closure mechanisms, prior intraocular surgery, and active ocular inflammation.

To avoid within-subject correlation, the primary analysis included one eye per patient. When bilateral eyes were included in sensitivity analyses, patient-clustered inference was applied (cluster-robust variance and/or subject-level cluster bootstrap).

### Interventions and treatment assignment

Participants underwent either Phaco-GSL (control group) or Phaco-GSL with adjunctive ab-interno goniotomy (intervention group). As this was a non-randomized study, treatment selection was determined by the attending surgeon based on preoperative assessment and intraoperative angle configuration. Confounding by indication was therefore expected. Causal adjustment methods were prespecified, but all causal estimates should be interpreted under measured-confounding assumptions, and residual confounding from unmeasured surgeon preference, medication response, optic nerve status, prior laser treatment, or intraoperative findings cannot be excluded.

All procedures were performed by experienced glaucoma specialists. Following standard phacoemulsification and intraocular lens implantation, goniosynechialysis (GSL) was performed using a viscoelastic device and micro-spatula to mechanically disengage PAS for 360° or to the maximal accessible extent. In the intervention group, an adjunctive ab-interno goniotomy was then performed, stripping the trabecular meshwork over approximately 120° of the nasal angle circumference to create a direct aqueous outflow bypass.

### Imaging acquisition and quantification

#### AS-OCT acquisition protocol

Preoperative AS-OCT was acquired using a standardized protocol. Imaging was performed in a dark room (<1 lux) to minimize physiologic angle widening due to light-induced miosis. Scans were obtained in manufacturer-recommended high-resolution mode, capturing nasal and temporal angles. Scleral spur visibility was verified, and scans without identifiable landmarks or with poor overall quality were excluded. Measurements were performed by trained graders masked to surgical outcomes. When repeated scans were available, the scan with the best quality grade was selected. All imaging-derived variables were strictly preoperative to prevent outcome leakage.

#### Quantitative parameters

Evaluated parameters included angle opening distance (AOD500 and AOD750), trabecular-iris space area (TISA500 and TISA750), trabecular-iris angle (TIA), anterior chamber depth (ACD), lens vault/lens convexity, trabecular meshwork reflectivity (grayscale intensity), iris thickness at 750 micrometers (IT750), iris curvature, plateau iris configuration (binary), and image quality grade. PAS extent was recorded in degrees based on gonioscopy. AS-OCT features were used for exploratory CATE and policy-learning models; ATE adjustment used the core clinical confounders specified below.

#### UBM acquisition

When clinically indicated and available, ultrasound biomicroscopy (UBM) was performed to evaluate ciliary body configuration and iris root anatomy using a standardized protocol (supine position, dark-room conditions<1 lux, sterile immersion eyecup with saline coupling, avoidance of excessive probe pressure). Radial scans centered at the limbus were obtained in four quadrants (12, 6, 3, 9 o’clock), with additional scans when plateau iris configuration or ciliary body abnormalities were suspected. Plateau iris configuration was defined as anteriorly positioned ciliary processes supporting a relatively flat central iris plane with a steep peripheral iris root, in the absence of pupillary block.

### Outcome measures

#### Primary endpoint

The primary endpoint was time-to-qualified-failure through 24 months. Qualified success was defined as IOP 5–18 mmHg and at least 20% reduction from baseline, with or without medications and without additional glaucoma surgery. Qualified failure was defined as loss of qualified success and/or the need for additional glaucoma surgery. In practical clinical terms, qualified failure meant that an eye no longer maintained target-range IOP control under the prespecified success criteria despite allowance for medications, or required further glaucoma surgery. Where failure criteria required confirmation, failure was defined on consecutive visits. This endpoint was selected because it captures durability of surgical control in routine glaucoma care, where postoperative medication resumption is common and medication-free complete success may be overly stringent.

#### Secondary endpoints

Secondary endpoints included: 1) Binary success outcomes at fixed time points, including 24-month medication-free complete success (IOP 5–18 mmHg with at least 20% reduction from baseline without IOP-lowering medications or additional glaucoma surgery), 24-month qualified success (the same IOP criteria with or without medications), and prespecified 12-month medication-free complete success in the development cohort. 2) Continuous postoperative outcomes, including IOP (mmHg) and number of IOP-lowering medications at prespecified follow-up visits. 3) Safety outcomes, including hyphema and IOP spikes (>30 mmHg).

### Causal framework, identification assumptions, and preprocessing

#### Potential outcomes and estimands

For each eye with baseline covariates, let denote treatment assignment (1 = Phaco-GSL + goniotomy; 0 = Phaco-GSL). Define potential outcomes and as the outcome under treated and untreated conditions, respectively. The marginal causal estimand for fixed-time outcomes was the average treatment effect (ATE), and effect variation was summarized via conditional average treatment effects (CATEs).


τ(x)=E[Y(1)−Y(0)∣X=x].

Causal identification relied on conditional exchangeability given measured covariates, positivity/overlap, and consistency.

#### Standardization and baseline missingness

All preprocessing and model-development decisions were defined *a priori* and learned within the development cohort. Continuous variables were inspected for implausible values and standardized (z-score) using development-cohort mean and standard deviation; for cross-validated analyses, preprocessing parameters were estimated within training folds and applied to held-out folds.


$$Z=\frac{X-\mu }{\sigma }.$$

Baseline covariate missingness was minimal. Among the core propensity-score covariates, only one PAS value was missing. The primary analysis used median imputation for continuous variables and mode imputation for categorical variables to preserve the consecutive cohort; imputation parameters were estimated within cross-validation folds for model training and evaluation. To address the concern that simple imputation might be incongruent with the otherwise advanced causal framework, we added a complete-case sensitivity analysis excluding the eye with missing PAS.

#### Propensity score modeling and weight diagnostics

The propensity score was estimated using logistic regression:


e(x)=Pr(T=1X=x).

For causal adjustment, covariates for the primary propensity score model were selected *a priori* according to three criteria: clinical plausibility as pre-treatment confounders of surgical selection and outcome, availability in structured records for nearly all eyes, and the need to limit model dimensionality in this small pilot cohort. The core model therefore included age, sex, baseline IOP, baseline medication number, PAS extent, ACD, and lens vault/convexity. These variables were chosen to represent demographic factors, baseline disease control, treatment intensity, synechial angle closure, and lens-related anterior-segment anatomy. Inverse probability of treatment weighting (IPTW) weights were constructed as (10, 11).


$$w_{i}=\frac{T_{i}}{\hat{e}\left(X_{i}\right)}+\frac{1-T_{i}}{1-\hat{e}\left(X_{i}\right)}.$$

Stabilized weights were additionally computed. Overlap was evaluated using propensity score distributions and extreme-weight diagnostics. Covariate balance was assessed using standardized mean differences (SMD), with SMD<0.10 considered acceptable. Extreme weights were mitigated in sensitivity analyses using truncation at the 99th percentile; propensity score trimming outside 0.05–0.95 was also evaluated (11).

Other clinically important factors may influence surgical selection, including functional glaucoma severity, optic nerve status, history of acute angle-closure attacks, prior laser peripheral iridotomy, medication response, and intraoperative angle findings after goniosynechialysis. Baseline visual field (VF) indices, including mean deviation (MD), pattern standard deviation (PSD), and Visual Field Index (VFI), and history of acute angle-closure attack were therefore evaluated in an expanded propensity-score sensitivity analysis. Prior laser status, medication response patterns, optic nerve imaging parameters, and detailed intraoperative angle findings were not consistently available in structured form and could not be included in the primary adjustment set; this limitation is explicitly acknowledged.

#### Fixed-time causal effects via doubly robust estimation

For binary and continuous endpoints at fixed time points, marginal causal effects were estimated using augmented inverse probability weighting (AIPW), combining the propensity score with outcome nuisance models (12). For clinical interpretation, AIPW combines propensity-score reweighting with outcome prediction; under standard measured-confounding assumptions, it can remain consistent when either the treatment-assignment model or the outcome model is adequately specified.


$${\hat{m}}_{1}\left(X\right)=E\left[YT=1,X\right],{\hat{m}}_{0}\left(X\right)=E\left[YT=0,X\right].$$

The AIPW pseudo-outcome (influence function) for the ATE was


$${\hat{\phi }}_{i}={\hat{m}}_{1}\left(X_{i}\right)-{\hat{m}}_{0}\left(X_{i}\right)+\frac{T_{i}\left\{Y_{i}-{\hat{m}}_{1}\left(X_{i}\right)\right\}}{\hat{e}\left(X_{i}\right)}-\frac{\left(1-T_{i}\right)\left\{Y_{i}-{\hat{m}}_{0}\left(X_{i}\right)\right\}}{1-\hat{e}\left(X_{i}\right)}.$$

The ATE was obtained by averaging ${\hat{\phi }}_{i}$ across eyes. For binary outcomes, effects were reported as risk differences (treated minus control); for continuous outcomes, effects were reported as mean differences (treated minus control). Robust (sandwich) variance estimators were used for confidence intervals, with nonparametric bootstrap used for policy-value uncertainty.

#### Time-to-event analysis: qualified failure

Using date-based time-to-event data, we defined time-to-qualified-failure as the elapsed time from surgery to the first chart-derived date at which an eye met the prespecified qualified failure criteria. Follow-up time was recorded in months since surgery (days/30.44), with eyes without failure right-censored at the last documented follow-up. As a sensitivity analysis, we repeated the Kaplan–Meier and Cox analyses using a visit-based event-time definition (1, 3, 6, 12, 18, and 24 months), assigning events to the confirming visit when consecutive confirmation was required (**Supplementary Figure 5**).

Failure-free survival was summarized using Kaplan-Meier curves and compared between groups using the log-rank test. To align survival analyses with the marginal causal estimand used elsewhere, we additionally reported stabilized IPTW-weighted Kaplan-Meier curves and fitted a stabilized IPTW-weighted Cox proportional hazards model with treatment as the sole covariate (Breslow handling of ties) using robust (sandwich) variance estimation to account for weighting.

#### Longitudinal postoperative IOP and medication trajectories

For repeated postoperative IOP and medication measurements, longitudinal marginal models included the 1-, 12-, 18-, and 24-month visits because these time points were consistently available as harmonized quantitative fields in the analytic dataset. The 3- and 6-month visits were used for visit-based qualified-failure sensitivity analyses when relevant, but they were not included in the continuous trajectory models because IOP and medication data at these intermediate visits were not uniformly structured across the cohort. We fit IPTW-weighted marginal models of the form:


Outcome∼C(Time)×Treatment,

where Time was treated as a categorical factor and Treatment indicated receipt of goniotomy. Models were estimated by weighted least squares using stabilized IPTW, with cluster-robust standard errors at the patient level to account for within-eye correlation over time. From the fitted models, we reported marginal treatment differences (Intervention − Control) at each time point with 95% confidence intervals.

#### Missing data handling

Binary success outcomes at 12 and 24 months were complete. For continuous postoperative outcomes (IOP and medication number), a small amount of missingness occurred at later follow-up visits (18 and 24 months). Analyses of continuous outcomes used complete-case data at each time point. As sensitivity analyses, longitudinal marginal models included all available repeated measurements under the assumption of missing at random conditional on observed history and stabilized IPTW, and the primary AIPW analysis was repeated after excluding the single eye with missing baseline PAS.

#### Benchmark strategy: phenotype-guided rule

To reflect pragmatic clinical reasoning, we prespecified a simple phenotype-guided strategy as an interpretable benchmark for exploratory comparisons. A high-risk phenotype was defined as PAS >=180 degrees or baseline medication burden >=3 agents. PAS >=180 degrees represents synechial closure over at least half of the angle circumference, whereas a medication burden of at least three agents reflects high preoperative treatment intensity and perceived risk of inadequate postoperative control. Under this benchmark strategy, high-risk eyes would be assigned adjunctive goniotomy and others would receive standard surgery. This strategy was evaluated as a hypothetical allocation rule rather than as a recommendation for clinical practice.

#### Exploratory CATE model: DR-learner and tau-hat-guided strategy (proof-of-concept)

Exploratory CATEs were estimated from baseline clinical and preoperative imaging covariates using a doubly robust learner (DR-learner) (9). Clinically, the DR-learner was introduced to address whether specific baseline anatomic and clinical profiles might derive greater absolute benefit from adding goniotomy. Briefly, we constructed an augmented inverse probability weighted (AIPW) pseudo-outcome using prespecified propensity score and outcome nuisance models fitted with logistic regression, and then regressed this pseudo-outcome on baseline covariates using ridge regression to obtain tau-hat. Tau-hat can be interpreted as a model-predicted absolute probability difference on the risk-difference scale for 24-month medication-free complete success; values greater than 0 indicate predicted benefit from adjunctive goniotomy, whereas values less than 0 indicate predicted lack of benefit or possible harm relative to Phaco-GSL alone. Highly flexible machine-learning learners were not used in the primary analyses given sample size. Because the DR-learner used ridge regression on an AIPW pseudo-outcome, predicted tau-hat values were not mathematically constrained to the theoretical risk-difference range of -1 to 1. Extreme predicted values were therefore not interpreted as literal individual probabilities. The CATE model was used only to explore whether baseline clinical and imaging features could generate signals of differential benefit, not to provide reliable patient-level surgical recommendations.

As a proof-of-concept illustration, we evaluated a simple prespecified tau-hat-guided strategy that recommends adjunctive goniotomy when the predicted tau-hat is nonnegative (tau-hat >= 0). This threshold was not optimized to the dataset and should not be interpreted as a clinically actionable cutoff. No tau-hat threshold was proposed for clinical use, and all tau-hat-guided policy analyses were treated as internal, hypothesis-generating demonstrations.

#### Exploratory internal policy and net benefit analysis

Detailed policy-value and net-benefit methods and internal results are provided in the Supplement (**Supplementary Table 8** and **Supplementary Figure 8**). Briefly, we compared treatment-all, treatment-none, phenotype-guided, and tau-hat-guided prototype strategies for 24-month medication-free complete success. Net benefit was evaluated across a grid of delta values, where delta represents the minimum acceptable absolute increase in success probability required to justify adding goniotomy. Because these comparisons were performed within the same development cohort used for model fitting and were not externally validated, they were treated as methodological illustrations rather than clinical allocation rules.


NB(π;δ)=(V(π)−V(treat-none))−δ·r(π).

#### Overfitting and stability analysis (10%-deletion refitting)

Given the limited sample size, we anticipated concerns regarding overfitting and the stability of tau-hat estimates. In addition to ridge penalization, we quantified the stability of the CATE model and the resulting tau-hat-guided recommendations using a prespecified resampling procedure. Specifically, we repeated 10%-deletion refitting: in each iteration, we randomly removed 10% of patients, refit the full DR-learner with identical specifications, and recomputed tau-hat and the tau-hat >= 0 recommendation for all eyes. We summarized the distribution of (i) treatment recommendation rates, (ii) agreement of recommendations with the full-cohort fitted model, and (iii) correlation of tau-hat predictions. This analysis is descriptive and intended to assess robustness rather than to provide additional confirmatory inference. Summary metrics are reported in **Supplementary Table 5**.

#### Sensitivity analyses

Prespecified sensitivity analyses included (1) propensity score trimming (0.05-0.95), (2) stabilized weights and 99th-percentile weight truncation, (3) complete-case analysis excluding the single eye with missing baseline PAS, and (4) an expanded propensity-score sensitivity analysis additionally including history of acute angle-closure attack, baseline VF MD, VF PSD, VF VFI, and plateau iris configuration. The expanded model was considered exploratory because of the limited sample size and increased number of covariates relative to the number of events.

#### Statistical software

Analyses were performed using R (v4.3.2; packages: survey, survival) and Python (scikit-learn, numpy, pandas). Two-sided p<0.05 was considered statistically significant.

## Results

### Study population and baseline characteristics

**Figure 2:**
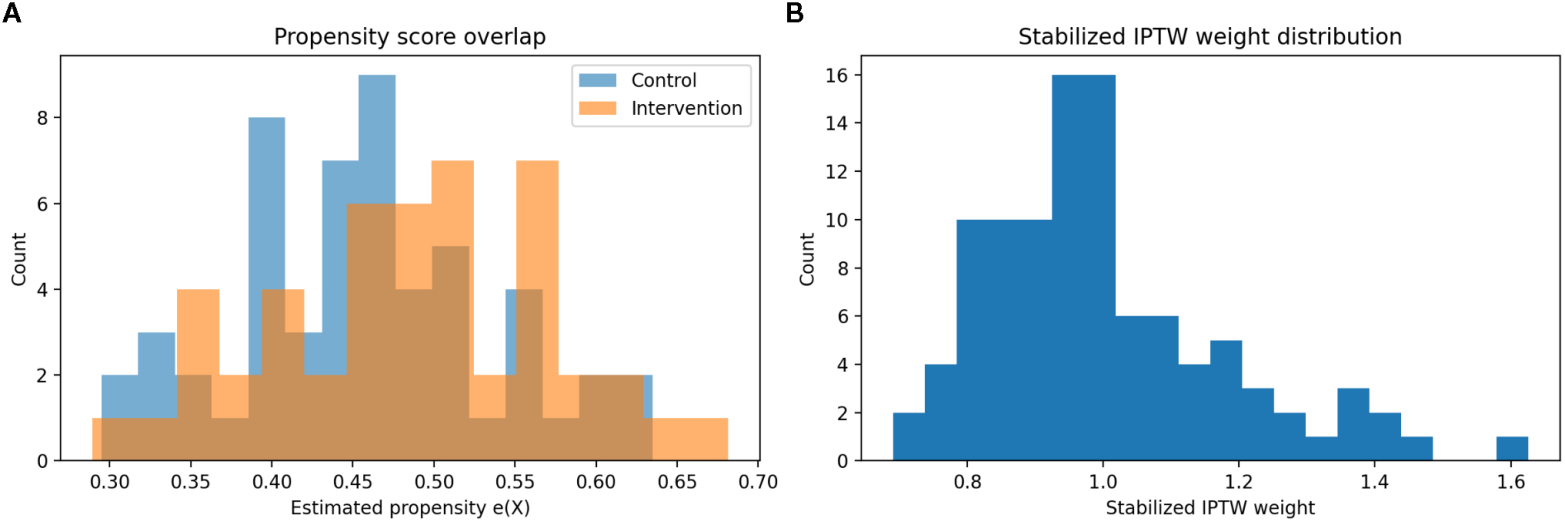
Propensity-score overlap and weight diagnostics (development cohort). **(A)** Estimated propensity score distributions (probability of receiving adjunctive ab-interno goniotomy) are shown by treatment group. Adequate overlap across groups supports the positivity/overlap assumption required for IPTW-based causal estimation; limited mass near 0 or 1 suggests a low risk of extreme extrapolation. **(B)** Distribution of stabilized inverse probability of treatment weights (IPTW). The absence of excessively large weights indicates acceptable variance inflation and reduces sensitivity to a small number of influential observations. Together, panels **(A, B)** summarize overlap and weight stability used to support the weighted estimators in subsequent analyses.

The development cohort included 102 eyes (one eye per patient): 54 underwent Phaco-GSL alone (control) and 48 underwent Phaco-GSL with adjunctive ab-interno goniotomy (intervention). Baseline characteristics are summarized in **Table 1**. Propensity score overlap was adequate (**Figure 2A**), and stabilized IPTW weight distributions demonstrated acceptable weight stability (**Figure 2B**). Stabilized IPTW weights achieved good covariate balance across the prespecified core confounders, with all post-weighting standardized mean differences below 0.10 (**Supplementary Figure 3**).

**Table 1:** Baseline characteristics of the development cohort.

Characteristic	Control (n=54)	Intervention (n=48)	Unweighted SMD
Age (years)	63.41 ± 9.12	64.56 ± 8.91	0.128
Baseline IOP (mmHg)	21.72 ± 2.82	22.12 ± 2.77	0.144
Baseline medications (n)	2.72 ± 0.68	2.85 ± 0.74	0.185
Peripheral anterior synechiae (PAS) extent (degrees)	164.89 ± 71.87	175.70 ± 61.73	0.161
Anterior chamber depth (mm)	2.83 ± 0.12	2.81 ± 0.15	-0.136
Lens convexity/vault (mm)	0.56 ± 0.11	0.57 ± 0.13	0.029
AOD500 (µm)	179.96 ± 43.57	183.27 ± 56.41	0.066
AOD750 (µm)	265.17 ± 49.99	261.10 ± 66.20	-0.069
TISA500 (mm²)	0.04 ± 0.01	0.04 ± 0.01	-0.341
TISA750 (mm²)	0.07 ± 0.02	0.07 ± 0.02	0.201
TIA (degrees)	18.67 ± 3.79	17.91 ± 3.44	-0.209
Trabecular meshwork reflectivity (grayscale)	134.91 ± 18.17	131.31 ± 15.61	-0.212
Iris thickness at 750 µm (µm)	521.48 ± 58.17	516.48 ± 58.40	-0.086
Iris curvature	0.20 ± 0.07	0.21 ± 0.06	0.282
Angular recess area (mm²)	0.04 ± 0.01	0.04 ± 0.01	-0.036
Ciliary body thickness (µm)	1120.09 ± 82.21	1126.75 ± 96.34	0.074
Time since last acute attack (weeks)	0.0 [0.0, 7.0]	0.0 [0.0, 0.0]	-0.092
Visual field (VF) mean deviation (MD) (dB)	-9.59 ± 1.46	-9.54 ± 1.53	0.034
Visual field pattern standard deviation (PSD) (dB)	5.05 ± 0.95	5.32 ± 0.83	0.302
Visual Field Index (VFI) (%)	59.55 ± 4.07	60.25 ± 5.22	0.148
Male sex	17/54 (31.5%)	17/48 (35.4%)	0.083
History of acute angle-closure attack	15/54 (27.8%)	11/48 (22.9%)	-0.112
Plateau iris configuration	10/54 (18.5%)	7/48 (14.6%)	-0.106
AS-OCT image quality: excellent	46/54 (85.2%)	37/48 (77.1%)	-0.207

Continuous variables are presented as mean ± standard deviation or median [interquartile range]. Categorical variables are presented as count/total (percentage). IOP, intraocular pressure; PAS, peripheral anterior synechiae; VF, visual field; MD, mean deviation; PSD, pattern standard deviation; VFI, Visual Field Index; SMD, standardized mean difference. SMDs in this table are unweighted baseline SMDs. Post-weighting balance for prespecified core confounders is shown in **Supplementary Figure 3**.

Stabilized IPTW showed limited variance inflation, with mean weight 1.000, SD 0.178, range 0.691-1.625, and 99th percentile 1.450. The overall effective sample size after weighting was approximately 98.9 eyes, with group-specific effective sample sizes of 52.7 in the control group and 46.2 in the intervention group (**Supplementary Table 2**). In the expanded propensity-score sensitivity analysis including history of acute angle-closure attack, baseline VF MD, VF PSD, VF VFI, and plateau iris configuration, weight behavior remained acceptable, with overall effective sample size approximately 95.2 eyes and maximum stabilized weight 2.25 (**Supplementary Table 7**).

#### Primary endpoint: Time-to-qualified-failure

**Figure 3:**
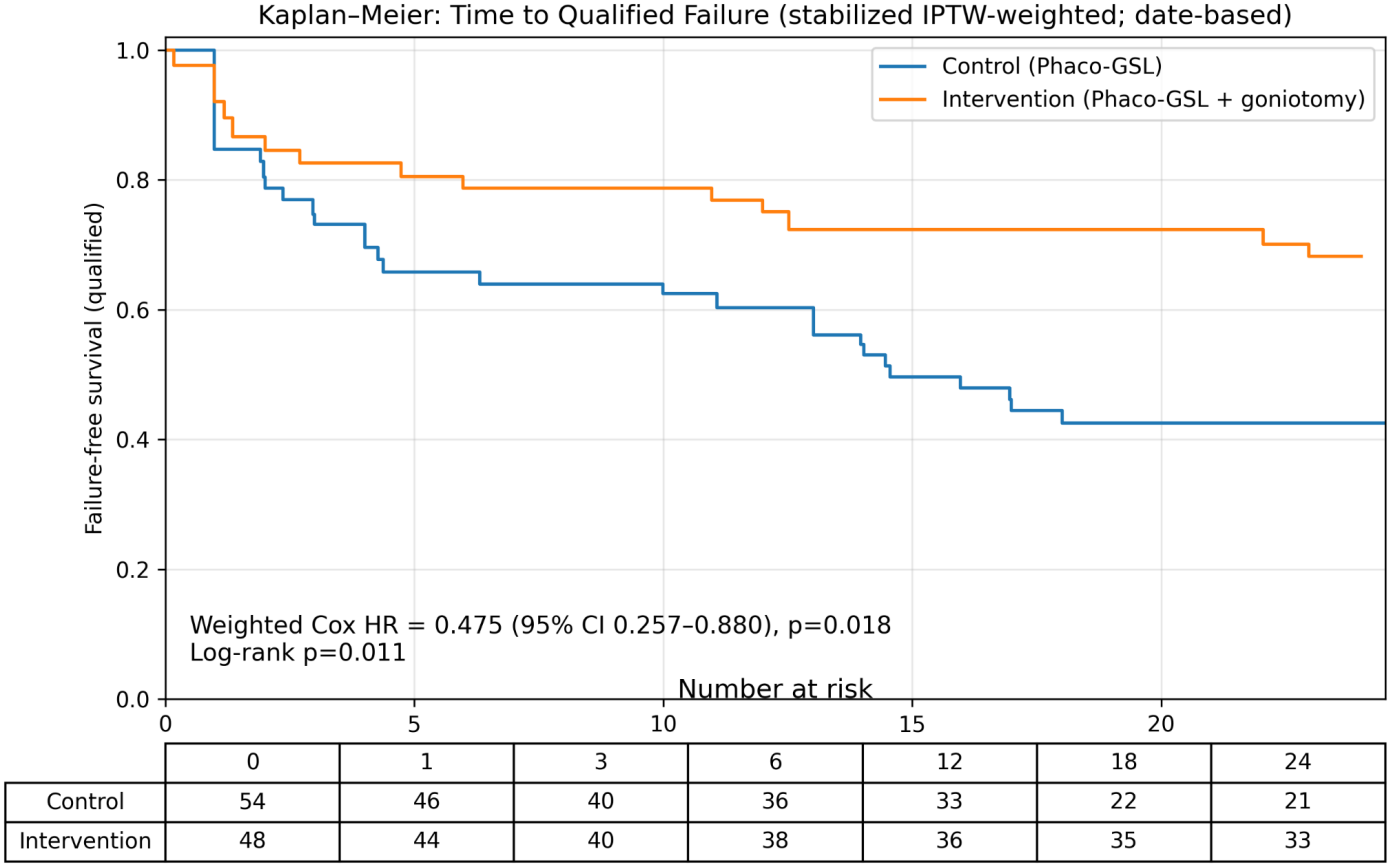
Kaplan-Meier curves for time to qualified failure (stabilized IPTW-weighted; date-based). Stabilized IPTW-weighted Kaplan-Meier estimates of qualified-failure-free survival over 24 months are shown for the control group (Phaco-GSL) and the intervention group (Phaco-GSL + ab-interno goniotomy). Time was calculated using surgery and event dates (date-based definition); participants without qualified failure were administratively censored at 24 months or censored at their last observed follow-up, as applicable. Numbers at risk are displayed below the curves. The hazard ratio (HR) and 95% confidence interval were obtained from a stabilized IPTW-weighted Cox model with treatment as the sole covariate; the annotated log-rank p-value summarizes group differences in weighted survival curves.

For the primary endpoint of time-to-qualified-failure, using date-based time-to-event data, adjunctive goniotomy was associated with higher qualified-failure-free survival. Across 24 months, 48 qualified-failure events were identified, including 33 events in the control group and 15 events in the intervention group. **Figure 3** shows the stabilized IPTW-weighted Kaplan-Meier curves. Qualified-failure-free survival at 24 months was 0.425 in the control group and 0.682 in the intervention group. The stabilized IPTW-weighted Cox proportional hazards model estimated a hazard ratio (HR) of 0.475 (95% CI 0.257 to 0.880; p=0.018). The unweighted log-rank test similarly favored the intervention (p=0.011). The corresponding event and censoring summary is provided in **Supplementary Table 4**.

#### Key secondary endpoints: binary success, continuous outcomes, and safety

Analysis of binary endpoints at fixed time points was consistent with the survival analysis. For 24-month medication-free complete success, augmented inverse probability weighting (AIPW) estimated an absolute risk difference of 0.291 (29.1 percentage points; 95% CI 0.105 to 0.478; p=0.002) favoring adjunctive goniotomy (**Table 2**). The 24-month qualified success endpoint also favored adjunctive goniotomy (risk difference 0.233; 95% CI 0.064 to 0.403; p=0.007). At 12 months, medication-free complete success similarly favored adjunctive goniotomy (risk difference 0.251; 95% CI 0.069 to 0.433; p=0.007). Complete-case sensitivity analysis excluding the eye with missing baseline PAS yielded a similar estimate for 24-month medication-free complete success (risk difference approximately 0.286).

**Table 2:** Development cohort AIPW estimates.

Outcome	Time	Effect type	Estimate	95% CI	P	N
Medication-free complete success	24 months	Risk difference	0.291	0.105 to 0.478	0.002	102
Qualified success	24 months	Risk difference	0.233	0.064 to 0.403	0.007	102
Medication-free complete success	12 months	Risk difference	0.251	0.069 to 0.433	0.007	102
Hyphema (any)	Period.	Risk difference	0.091	-0.002 to 0.185	0.056	102
IOP (mmHg)	12 months	Mean difference	-1.912	-3.020 to -0.803	0.001	102
Medication number	12 months	Mean difference	-0.655	-1.091 to -0.218	0.003	102
IOP (mmHg)	24 months	Mean difference	-1.697	-2.869 to -0.525	0.005	98
Medication number	24 months	Mean difference	-0.438	-0.861 to -0.015	0.042	98

Effect estimates are marginal augmented inverse probability weighting (AIPW) average treatment effects. For binary outcomes, the effect estimate is a risk difference (treated minus control); for continuous outcomes, the effect estimate is a mean difference (treated minus control). IOP, intraocular pressure.

Continuous outcomes were analyzed with AIPW using complete-case data at each time point. The average between-group IOP difference was modest. At 12 months, adjunctive goniotomy was associated with lower IOP by -1.91 mmHg on average (95% CI -3.02 to -0.80; p=0.001) and fewer medications by -0.65 agents (95% CI -1.09 to -0.22; p=0.003). At 24 months, analyses included 98 eyes with observed 24-month IOP/medication values; adjunctive goniotomy was associated with lower IOP by -1.70 mmHg (95% CI -2.87 to -0.52; p=0.005) and fewer medications by -0.44 agents (95% CI -0.86 to -0.02; p=0.042) (**Table 2**).

Regarding safety, hyphema occurred numerically more often in the intervention group. In the development cohort, any perioperative hyphema was numerically more common with adjunctive goniotomy (risk difference 0.091; 95% CI -0.002 to 0.185; p=0.056) (**Table 2**).

### Longitudinal postoperative trajectories

**Figure 4:**
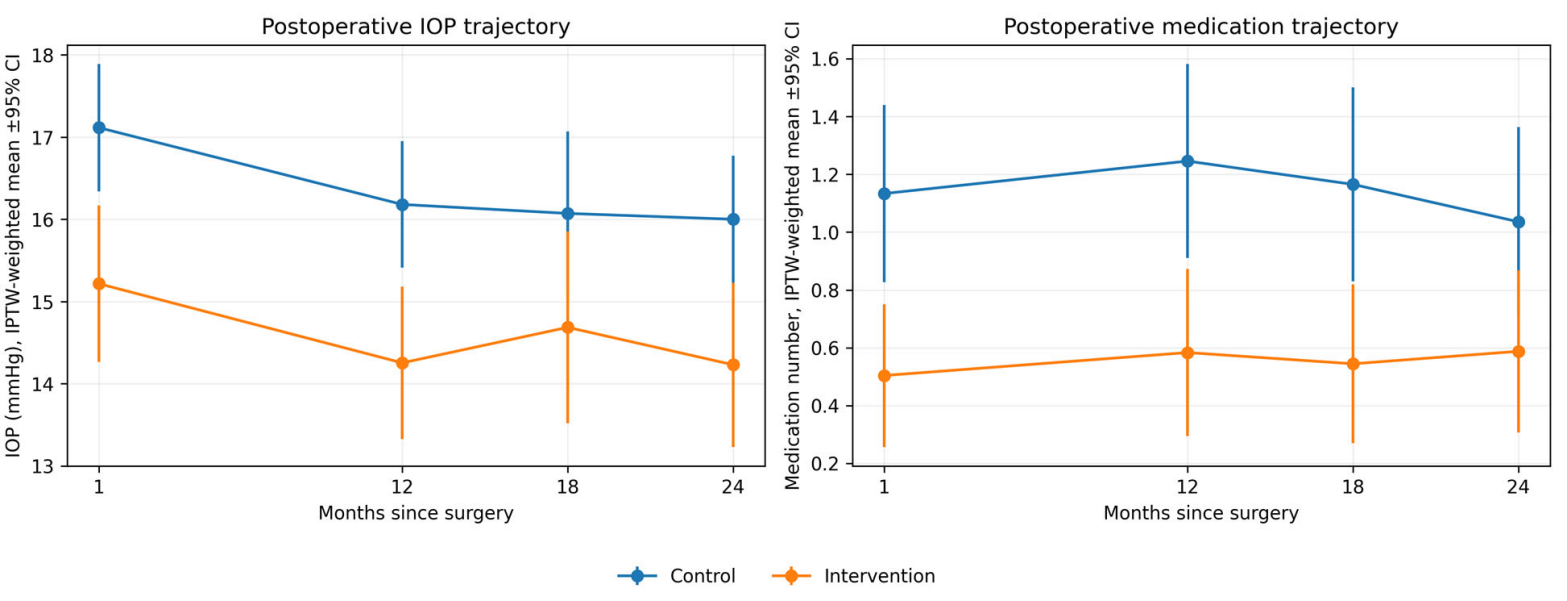
Model-estimated longitudinal trajectories of IOP and medication burden. Marginal mean trajectories of intraocular pressure (IOP, mmHg) and medication burden (number of topical agents) are shown at prespecified postoperative time points, estimated from stabilized IPTW-weighted marginal models to account for baseline confounding. Points represent weighted marginal means, and vertical bars denote 95% confidence intervals. These trajectories summarize average postoperative profiles by treatment group under the causal model used for marginal effect estimation.

**Figure 4** presents model-estimated longitudinal trajectories for IOP and medication burden. Values represent marginal means (95% CI) derived from stabilized IPTW-weighted marginal models with a time-by-treatment interaction and patient-level cluster-robust standard errors. Across follow-up, the intervention group maintained lower IOP and required fewer medications than the control group, consistent with the fixed-time AIPW estimates (**Table 2**).

### Exploratory CATE analysis and prototype strategy evaluation

**Figure 5:**
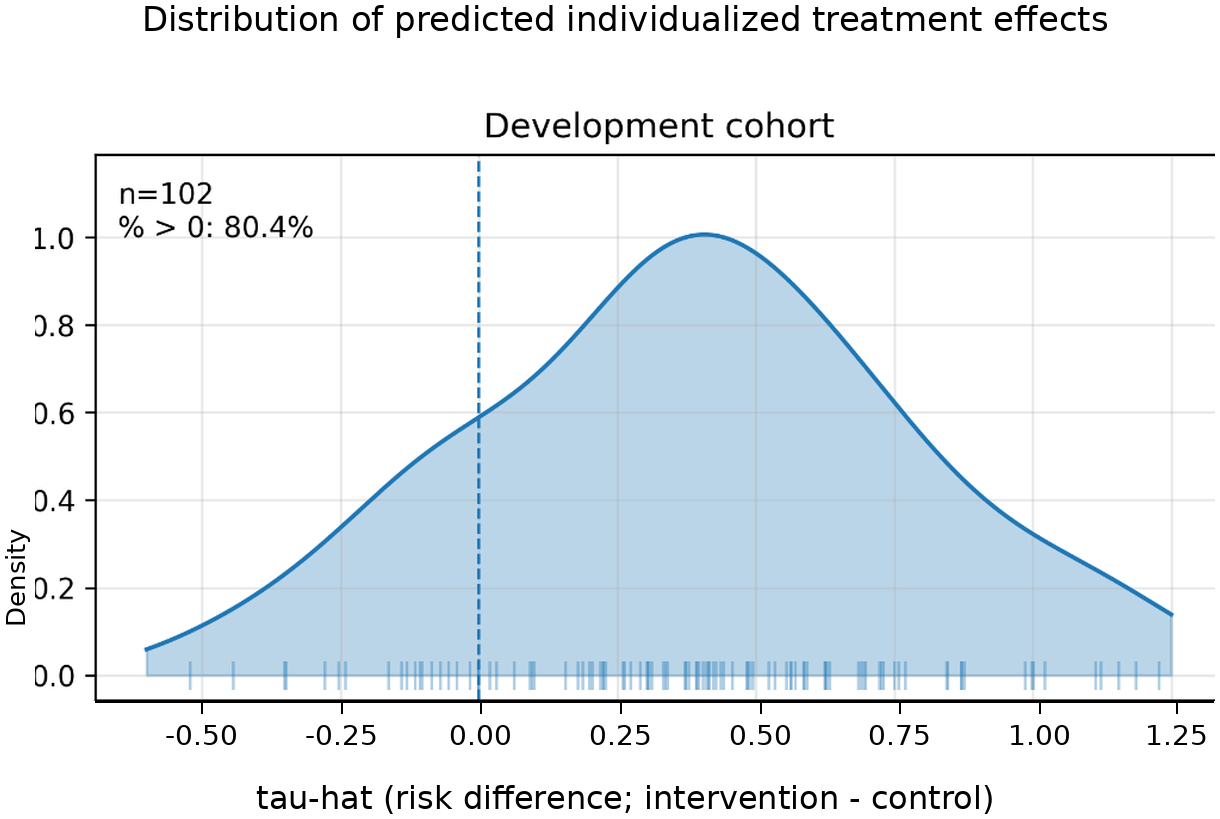
Distribution of predicted individualized benefit estimates (tau-hat) on the risk-difference scale (intervention - control). Kernel density estimates of predicted individualized benefit estimates (tau-hat) are shown for the development cohort, with rug plots indicating individual observations. Tau-hat represents the model-predicted CATE estimate on the risk-difference scale (intervention minus control) for 24-month medication-free complete success; values >0 indicate predicted benefit from adjunctive goniotomy, whereas values<0 suggest potential lack of benefit or harm relative to control. The dashed vertical line denotes tau-hat = 0, highlighting the decision boundary used in prototype policy analyses. The dispersion of tau-hat illustrates variation in predicted benefit.

The exploratory DR-learner produced variable tau-hat estimates (model-predicted CATE estimates; risk difference, intervention minus control) from baseline clinical and imaging features (**Figure 5**). Predicted tau-hat values showed dispersion compatible with possible variation in CATEs (range -0.70 to 1.33; median 0.30), but they were not considered clinically reliable patient-level recommendations. In particular, the ridge regression model fitted to the AIPW pseudo-outcome was unconstrained and generated some out-of-bound tau-hat values; extreme values were therefore not interpreted as literal patient-level probabilities. The small development cohort also limited calibration and external validity. Calibration across tau-hat strata was imperfect and nonmonotonic in this small development cohort, reinforcing that tau-hat should be interpreted only as an exploratory CATE signal rather than a calibrated patient-level benefit prediction (**Supplementary Table 3**). Because this cohort did not support stable interpretation of individual feature weights, AS-OCT feature-level signals were treated as hypotheses for future validation rather than as clinical predictors.

As an internal proof-of-concept analysis, we compared hypothetical treatment-all, treatment-none, phenotype-guided, and tau-hat-guided strategies. Because these policy comparisons were performed in the same development cohort used for model fitting and were not externally validated, detailed policy-value and net-benefit results were moved to the Supplement (**Supplementary Table 8**; **Supplementary Figure 8**). These results should be interpreted as methodological illustrations rather than evidence supporting a clinical allocation rule.

#### Stability analysis (addressing overfitting concerns)

In 10%-deletion refitting (300 iterations), the tau-hat >= 0 recommendation showed reasonable internal reproducibility but did not constitute validation. Median agreement with the full-cohort recommendations was 0.922 (IQR 0.902-0.951; 5th-95th percentile 0.863-0.971), and tau-hat predictions were correlated with full-cohort tau-hat (median Pearson r=0.907; 5th-95th percentile 0.825-0.957). However, 14/102 eyes (13.7%) showed moderate instability (recommended in 20-80% of refits), indicating uncertainty for cases near the tau-hat = 0 boundary. This internal stability check does not replace external validation.

## Discussion

### Principal findings

In this single-center proof-of-concept pilot cohort, we evaluated an AS-OCT-informed causal framework for estimating average treatment effects and exploring CATEs for adjunctive ab-interno goniotomy added to phacoemulsification with goniosynechialysis (Phaco-GSL) in chronic angle-closure glaucoma (CACG). The analytical approach combined propensity-score weighting and doubly robust estimation to address measured confounding in this nonrandomized surgical cohort (10–12, 26). Under the assumptions of measured-confounder exchangeability, positivity, and consistency, adjunctive goniotomy was associated with a lower risk of qualified failure through 24 months (stabilized IPTW-weighted HR, 0.48; 95% CI, 0.26–0.88) and a higher probability of 24-month medication-free complete success (AIPW risk difference, 0.29; 95% CI, 0.11–0.48). These associations were more apparent for failure-free surgical control and medication-free complete success than for average IOP reduction, which was modest. The findings should be interpreted as hypothesis-generating rather than practice-changing because treatment assignment was not randomized and the development cohort was small. The main contribution of this study is therefore methodological and exploratory: it demonstrates a transparent causal-inference workflow for estimating marginal surgical effects and for generating AS-OCT-informed hypotheses about CATEs in future larger studies (27).

### Clinical interpretation of the effect pattern

The observed effect pattern is biologically plausible in CACG. In eyes with long-standing synechial angle closure, anatomical reopening after Phaco-GSL may not fully restore trabecular and distal outflow function because chronic angle closure can be accompanied by persistent structural and functional outflow impairment (28). Adjunctive ab-interno goniotomy may therefore provide incremental benefit by bypassing or removing a segment of high-resistance trabecular tissue after goniosynechialysis, potentially improving the durability of postoperative IOP control beyond Phaco-GSL alone (17). Importantly, however, the average between-group IOP differences in the present cohort were modest, approximately 1.7–1.9 mmHg at 12–24 months. Thus, these data should not be interpreted as evidence that adjunctive goniotomy produces a large uniform IOP-lowering effect in all CACG eyes. Rather, the more clinically relevant signal was the higher probability of maintaining target-range IOP with fewer or no medications and the lower risk of qualified failure. One possible explanation is that goniotomy may improve access to conventional outflow and reduce the need to reintroduce medications or manage intermittent pressure elevations, whereas mean IOP reduction may remain constrained by distal outflow resistance and episcleral venous pressure. If confirmed prospectively, this pattern would suggest that the incremental value of adjunctive goniotomy may lie more in improving the durability of surgical control and reducing postoperative treatment burden than in producing large additional mean IOP reductions (14). Whether these differences translate into slower visual-field progression, improved adherence, reduced ocular-surface toxicity, or better quality of life requires longer prospective follow-up.

### Safety considerations

Safety findings should be interpreted in the context of the additional angle procedure. Hyphema occurred numerically more often after adjunctive goniotomy, consistent with the expected risk profile of trabecular meshwork incision or excision during angle surgery (29). In this pilot cohort, the estimated increase in any perioperative hyphema was approximately 9 percentage points, but the confidence interval included no difference, reflecting limited precision. No safety signal in this dataset was sufficient to outweigh the observed associations with qualified-failure-free survival and medication-free complete success; however, the study was underpowered to define uncommon complications or to quantify patient-level risk-benefit trade-offs. Larger prospective studies should use standardized adverse-event definitions and should incorporate complication severity, duration, and clinical consequence when evaluating whether the incremental benefit of adjunctive goniotomy justifies the additional procedure (30, 31).

### Exploratory CATEs

A secondary aim of this study was to explore whether baseline clinical and AS-OCT features could generate signals of CATE variation. The DR-learner was used to estimate exploratory CATEs for 24-month medication-free complete success, not to provide patient-level surgical recommendations. The observed dispersion of tau-hat values suggests that the incremental benefit of adjunctive goniotomy may not be uniform across CACG eyes; however, this finding must be interpreted cautiously. The development cohort was small, the model was internally evaluated rather than externally validated, and the ridge regression fitted to the AIPW pseudo-outcome was not constrained to the theoretical risk-difference range. Therefore, extreme tau-hat values should not be interpreted as literal individual probabilities. Moreover, correlated AS-OCT parameters such as AOD, TISA, ACD, and lens vault can yield unstable individual feature coefficients in small penalized models; therefore, we deliberately avoided interpreting feature weights as clinical predictors. Although cross-fitting, ridge penalization, and 10%-deletion refitting were used to reduce overfitting concerns, these procedures do not substitute for independent validation or calibration assessment. The policy and net-benefit analyses are therefore presented only as supplementary methodological illustrations. They should be viewed as a framework for designing future prospective studies rather than as evidence supporting a clinical allocation rule (32, 33).

## Limitations and future directions

Several limitations should define the interpretation of this study. First, treatment assignment was not randomized but determined by the attending surgeon. The decision to add goniotomy may have been influenced by factors that were incompletely captured in the dataset, including surgeon preference, perceived trabecular meshwork damage, medication response history, prior laser treatment, optic nerve status, and intraoperative angle appearance after goniosynechialysis. Propensity-score weighting and doubly robust estimators can reduce bias from measured confounders, but they cannot eliminate bias from unmeasured or poorly measured factors. Therefore, all causal interpretations depend on the assumptions of conditional exchangeability, positivity, and consistency.

Second, this was a single-center retrospective pilot cohort with a limited sample size and a modest number of qualified-failure events. Although stabilized IPTW showed acceptable weight behavior and the effective sample size was largely preserved after weighting, small-sample variability remains an important concern. The expanded propensity-score and complete-case sensitivity analyses supported the direction of the primary findings, but they do not substitute for replication in larger cohorts.

Third, the CATE and policy analyses are intrinsically exploratory. The DR-learner was developed and evaluated within the same cohort, and no external validation or prospective calibration assessment was available. The 10%-deletion refitting analysis provided an internal stability check, but it does not establish transportability across surgeons, imaging devices, surgical techniques, or patient populations. For this reason, tau-hat estimates and threshold-based policy simulations should not be used to guide clinical treatment selection.

Fourth, several clinically relevant outcomes were not available over a sufficiently long horizon. The present study focused on 24-month surgical success, IOP control, medication burden, and perioperative safety. It could not determine whether the observed differences translate into slower visual-field progression, reduced ocular-surface toxicity, improved adherence, better quality of life, or lower long-term need for additional glaucoma surgery. Future prospective multicenter studies should use standardized imaging protocols, harmonized definitions of medication-free complete success, qualified success, qualified failure, and adverse events, and prespecified causal and CATE analyses. Such studies should evaluate both average treatment effects and calibrated CATE estimates before AS-OCT-informed surgical allocation strategies are considered for clinical use (33, 34). Future studies could also test prespecified AS-OCT phenotypes as potential groups with larger absolute benefit from adjunctive goniotomy, such as eyes with pronounced lens-vault/anterior-chamber crowding but sufficient residual angle anatomy to permit effective trabecular access.

## Conclusions

In this retrospective single-center pilot cohort, adjunctive ab-interno goniotomy during Phaco-GSL was associated with more favorable 24-month qualified-failure-free survival and higher medication-free complete success in CACG. The average IOP difference was modest, suggesting that the potential incremental value of adjunctive goniotomy may relate more to durability of surgical control and reduced postoperative treatment burden than to large uniform IOP reduction. This study also demonstrates the feasibility of an AS-OCT-informed causal framework for estimating average treatment effects and exploring CATEs. However, the CATE estimates and policy simulations remain hypothesis-generating and require external validation before clinical use.

## Data Availability

The raw data supporting the conclusions of this article will be made available by the authors, without undue reservation.
