# Supplementary material for: Adjunctive ab-interno goniotomy in chronic angle-closure glaucoma: a retrospective proof-of-concept pilot study using doubly robust learning
Source: Front Ophthalmol (Lausanne). 2026 Jun 19;6:1846121. doi: 10.3389/fopht.2026.1846121 (PMC13327950; doi:10.3389/fopht.2026.1846121)

# Supplementary Material

This document provides expanded supplementary methods, diagnostics, sensitivity analyses, reproducibility details, and additional figures/tables supporting the main manuscript. Exploratory conditional average treatment effects (CATEs), summarized as predicted individualized benefit estimates (tau-hat), and policy analyses are presented as internal proof-of-concept analyses and are not intended for clinical implementation.

## Supplementary Methods

Propensity score diagnostics and covariate balance

Propensity scores were estimated by logistic regression with the prespecified core confounders (age, sex, baseline IOP, baseline medication number, peripheral anterior synechiae (PAS) extent, anterior chamber depth, and lens vault/convexity). These covariates were selected a priori because they were clinically plausible pre-treatment confounders, consistently available in structured records, and appropriate for the dimensionality of this pilot cohort. Stabilized IPTW weights were constructed and used throughout marginal and survival analyses. Balance was assessed using standardized mean differences (SMD); |SMD|<0.10 was considered acceptable. Effective sample size (ESS) was calculated as (sum w)^2 / sum(w^2), overall and within treatment groups.

An expanded propensity-score sensitivity model additionally included history of acute angle-closure attack, baseline visual field (VF) mean deviation (MD), VF pattern standard deviation (PSD), VF Visual Field Index (VFI), and plateau iris configuration. This expanded model was considered exploratory because of the limited sample size and increased number of covariates. Prior laser status, medication response patterns, optic nerve imaging parameters, and detailed intraoperative angle findings were not consistently available in structured form and therefore could not be included.

DR-learner CATE and tau-hat estimation and calibration

Predicted individualized benefit estimates (tau-hat) were obtained from a doubly robust learner (DR-learner) as a proof-of-concept CATE model: (i) nuisance propensity and outcome models were fit with logistic regression; (ii) an AIPW pseudo-outcome was constructed for 24-month medication-free complete success; and (iii) tau-hat was estimated by ridge regression of the pseudo-outcome on baseline clinical and AS-OCT features with 5-fold cross-fitting. Tau-hat represents a model-predicted absolute probability difference on the risk-difference scale. Because the model regressed an unconstrained pseudo-outcome, tau-hat predictions were not restricted to the theoretical risk-difference interval of -1 to 1. Extreme tau-hat values were not interpreted as literal individual probabilities. Tau-hat calibration was assessed by comparing mean predicted tau-hat to observed IPTW risk differences within strata. Calibration across tau-hat strata was imperfect and nonmonotonic in this small development cohort; therefore, tau-hat should be interpreted as an exploratory CATE signal rather than a calibrated patient-level benefit prediction. Given the limited sample size, stability was assessed via 10%-deletion refitting (Table S5).

Reproducibility details: all treatment, outcome, and imaging covariates used for the propensity, AIPW, and DR-learner analyses were strictly preoperative for treatment-assignment and CATE modeling. Continuous covariates were standardized using development-cohort means and standard deviations, with preprocessing performed within cross-validation folds for cross-fitted analyses. Baseline missingness was minimal; only one core propensity-score covariate value (PAS extent) was missing. The primary analysis used median/mode imputation, and a complete-case sensitivity analysis excluding this eye was added. Bootstrap uncertainty for the main AIPW sensitivity table used percentile intervals; policy analyses used nonparametric bootstrap resampling and are presented only as internal methodological illustrations. Feature-level interpretation of individual AS-OCT coefficients was not attempted in this pilot cohort because of limited sample size and correlation among angle-configuration variables.

## Supplementary Tables

Table S1. Sensitivity analyses for 24-month medication-free complete success (AIPW ATE on risk difference scale).

| Analysis | ATE (RD) | Bootstrap 95% CI | N |
| --- | --- | --- | --- |
| Main (stabilized) | 0.291 | 0.117 to 0.478 | 102 |
| Unstabilized | 0.291 | 0.097 to 0.465 | 102 |
| 99th pct trunc | 0.291 | 0.084 to 0.472 | 102 |
| PS trimmed 0.05-0.95 | 0.291 | 0.086 to 0.480 | 102 |

Binary ATEs are reported as risk differences (treated minus control). Bootstrap percentile CIs use 300 resamples. Additional complete-case and expanded-propensity sensitivity diagnostics are summarized in Table S7.

Table S2. Stabilized IPTW diagnostics (development cohort).

| Mean | SD | Min | P5 | P50 | P95 | P99 | Max |
| --- | --- | --- | --- | --- | --- | --- | --- |
| 1.000 | 0.178 | 0.691 | 0.773 | 0.970 | 1.361 | 1.450 | 1.625 |

Note: No extreme weights were observed; the maximum stabilized weight was 1.625. The overall effective sample size after weighting was 98.9 eyes, with group-specific effective sample sizes of 52.7 in the control group and 46.2 in the intervention group.

Table S3. Tau-hat calibration: predicted tau-hat versus observed IPTW risk difference (24-month medication-free complete success).

| Stratum | N | Mean predicted tau-hat | Observed IPTW RD |
| --- | --- | --- | --- |
| S1 | 19 | -0.262 | 0.168 |
| S2 | 21 | 0.000 | 0.188 |
| S3 | 21 | 0.089 | 0.485 |
| S4 | 20 | 0.273 | 0.463 |
| S5 | 21 | 0.675 | 0.226 |

Note: S1 includes tau-hat < 0. S2-S5 are quartiles among tau-hat >= 0. Observed effects are within-stratum stabilized IPTW risk differences. Calibration across strata was imperfect and nonmonotonic, reinforcing that these estimates are exploratory and not calibrated for patient-level decision-making.

Table S4. Primary date-based time-to-qualified-failure event and censoring summary.

| **Group** | **Qualified-failure events** | **Censored before 24 months** | **Administratively censored at 24 months** | **Total eyes** |
| --- | --- | --- | --- | --- |
| Control | 33 | 1 | 20 | 54 |
| Intervention | 15 | 0 | 33 | 48 |
| Overall | 48 | 1 | 53 | 102 |

Note: The primary time-to-event analysis used chart-derived date-based failure times; eyes without qualified failure were censored at last follow-up or administratively at 24 months.

Table S5. Stability analysis of tau-hat-guided recommendations under 10%-deletion refitting (development cohort).

| Metric | Median (IQR) | 5th-95th percentile / n (%) |
| --- | --- | --- |
| Recommendation rate (tau-hat >= 0) | 0.775 (0.755-0.794) | 0.716-0.824 |
| Agreement with full-cohort recommendations | 0.922 (0.902-0.951) | 0.863-0.971 |
| Pearson correlation with full-cohort tau-hat | 0.907 (0.881-0.930) | 0.825-0.957 |
| Eyes recommended in ≥90% of refits |  | 62/102 (60.8%) |
| Eyes recommended in ≤10% of refits |  | 11/102 (10.8%) |
| Eyes with moderate instability (20-80%) |  | 14/102 (13.7%) |

Note: 300 iterations were performed; each iteration removed 10 patients (~10%) and refit the prespecified DR-learner. Summary statistics are computed across refits. Note: The median recommendation rate across 10%-deletion refits was 77.5% (IQR, 75.5%-79.4%), whereas the full-cohort empirical selection rate for the tau-hat >= 0 strategy was 81.4%, as reported in Table S8.

Table S6. Stepwise analytical workflow used in the main manuscript.

| **Step** | **Purpose** | **Method** | **Output** |
| --- | --- | --- | --- |
| 1 | Define cohort and endpoints | Consecutive retrospective cohort; one eye per patient | 102 eyes; 24-month outcomes |
| 2 | Address treatment-selection bias | Logistic propensity score; stabilized IPTW | Overlap, SMD, weight diagnostics, ESS |
| 3 | Estimate average effects | IPTW Kaplan-Meier/Cox; AIPW fixed-time estimators | HRs, risk differences, mean differences |
| 4 | Explore CATEs / predicted individualized benefit | DR-learner with ridge regression and 5-fold cross-fitting | Exploratory predicted individualized benefit (tau-hat) distribution |
| 5 | Assess robustness | Weight truncation, PS trimming, complete-case analysis, expanded PS model | Sensitivity/stability metrics |
| 6 | Illustrate decision analysis | Internal policy and net-benefit simulation | Proof-of-concept only; not a clinical rule |

Table S7. Additional diagnostics and sensitivity analyses added in response to reviewer concerns.

| **Analysis** | **Key result** | **Interpretation** |
| --- | --- | --- |
| Primary stabilized IPTW | Mean 1.000; SD 0.178; range 0.691-1.625; P99 1.450; overall ESS 98.9 | Limited variance inflation; no extreme weights |
| Group-specific ESS | Control 52.7; intervention 46.2 | Effective sample size remained close to the original group sizes |
| Expanded PS model | Added acute attack history, VF MD, VF PSD, VF VFI, and plateau iris; overall ESS 95.2; max weight 2.25 | Weight behavior remained acceptable after adding severity/history covariates |
| Complete-case baseline analysis | Excluded the single eye with missing PAS; AIPW RD for 24-month medication-free complete success approximately 0.286 | Primary result was not driven by simple imputation |

Table S8. Exploratory internal strategy value comparison (bootstrapped 95% CI; proof-of-concept).

| Strategy | Treat rate (development cohort) | Value for 24-month medication-free complete success |
| --- | --- | --- |
| Treat-none | 0.0% | 0.349 (0.224, 0.486) |
| Phenotype | 78.4% | 0.656 (0.524, 0.787) |
| Tau-hat-guided (tau-hat >= 0) | 81.4% | 0.653 (0.505, 0.792) |
| Treat-all | 100.0% | 0.638 (0.493, 0.779) |

Note: Strategy value comparisons are based on hypothetical allocation strategies and internal evaluation within the same cohort used for model development. They are presented for methodological proof-of-concept and should not be interpreted as clinical recommendations.

## Supplementary Figures

Figure S1. Propensity score overlap by treatment group (development cohort).


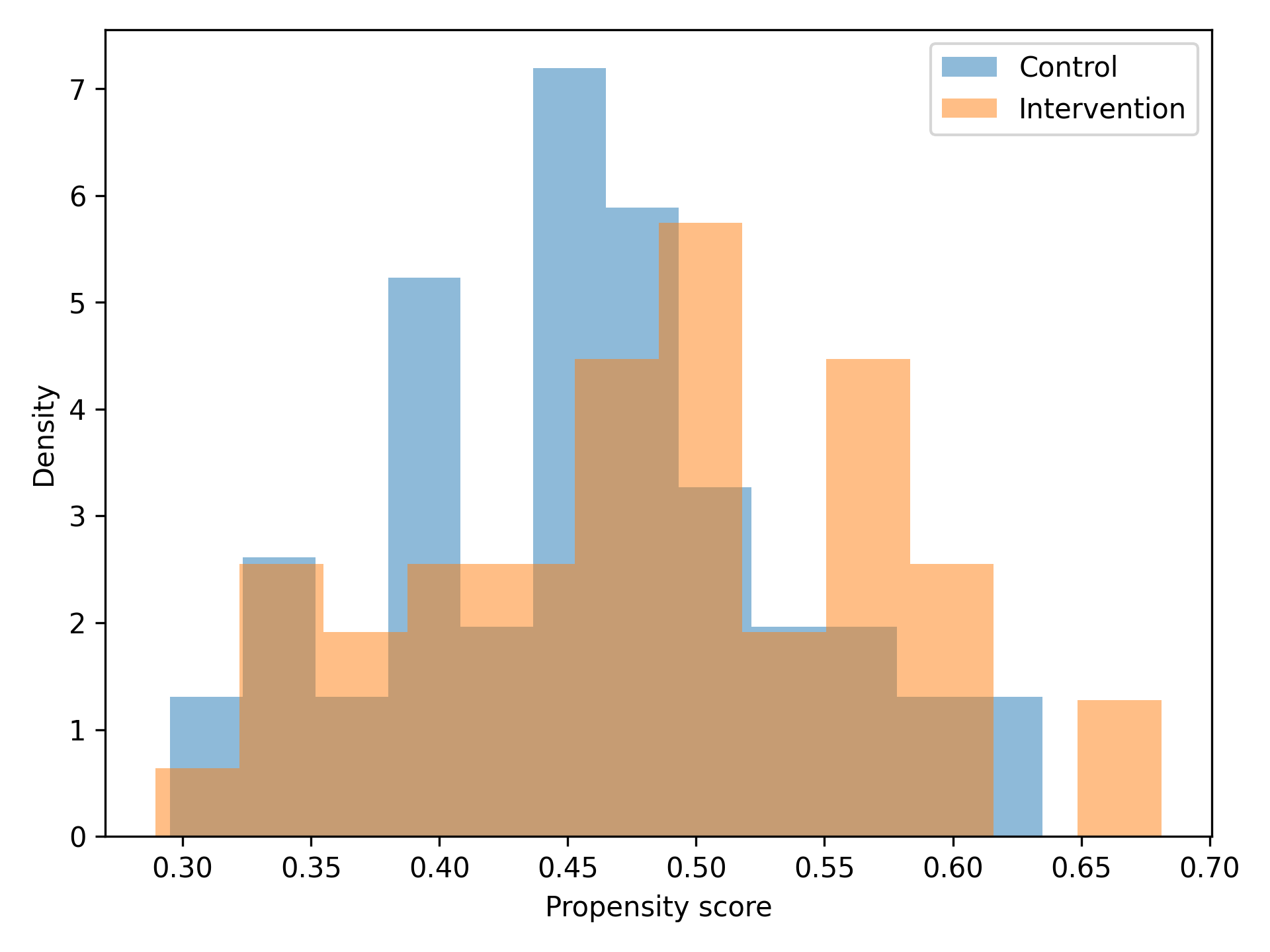


Figure S2. Distribution of stabilized IPTW weights (development cohort).


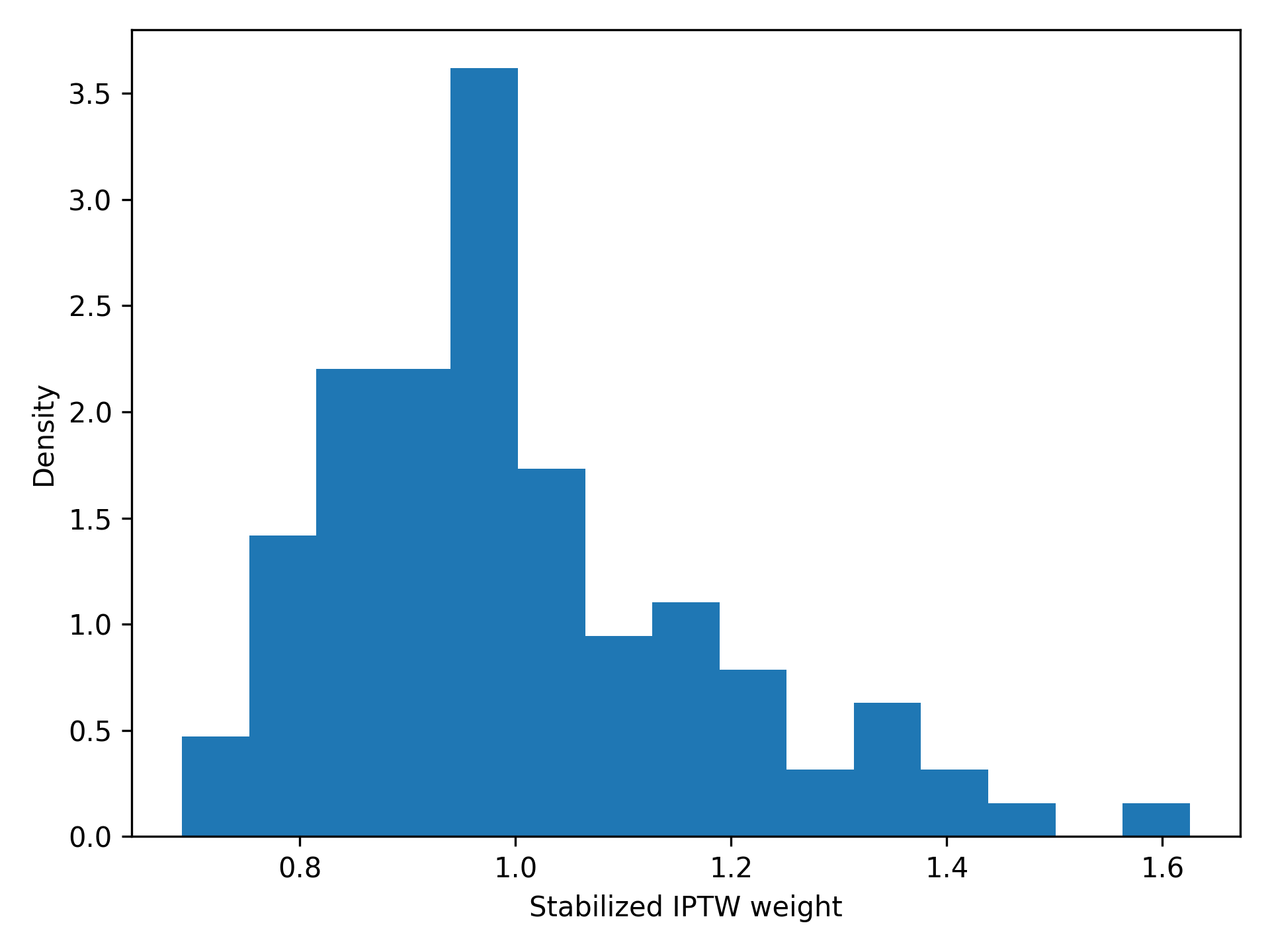


Figure S3. Covariate balance before and after stabilized IPTW (Love plot) for prespecified core confounders.


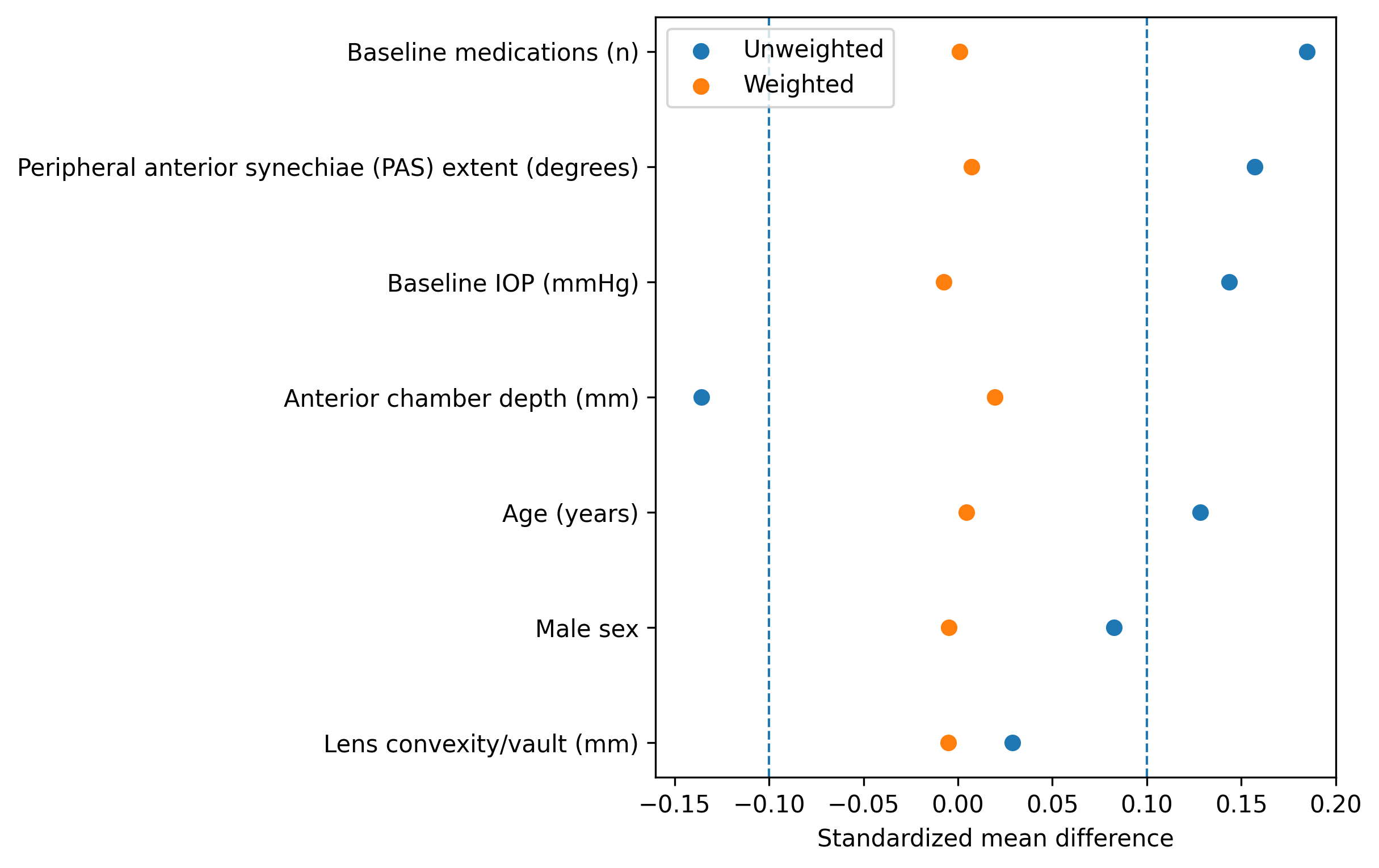


Figure S4. Primary date-based Kaplan-Meier curve for time-to-qualified-failure (stabilized IPTW-weighted), with numbers at risk. This figure uses the date-based event-time definition used in the main analysis.


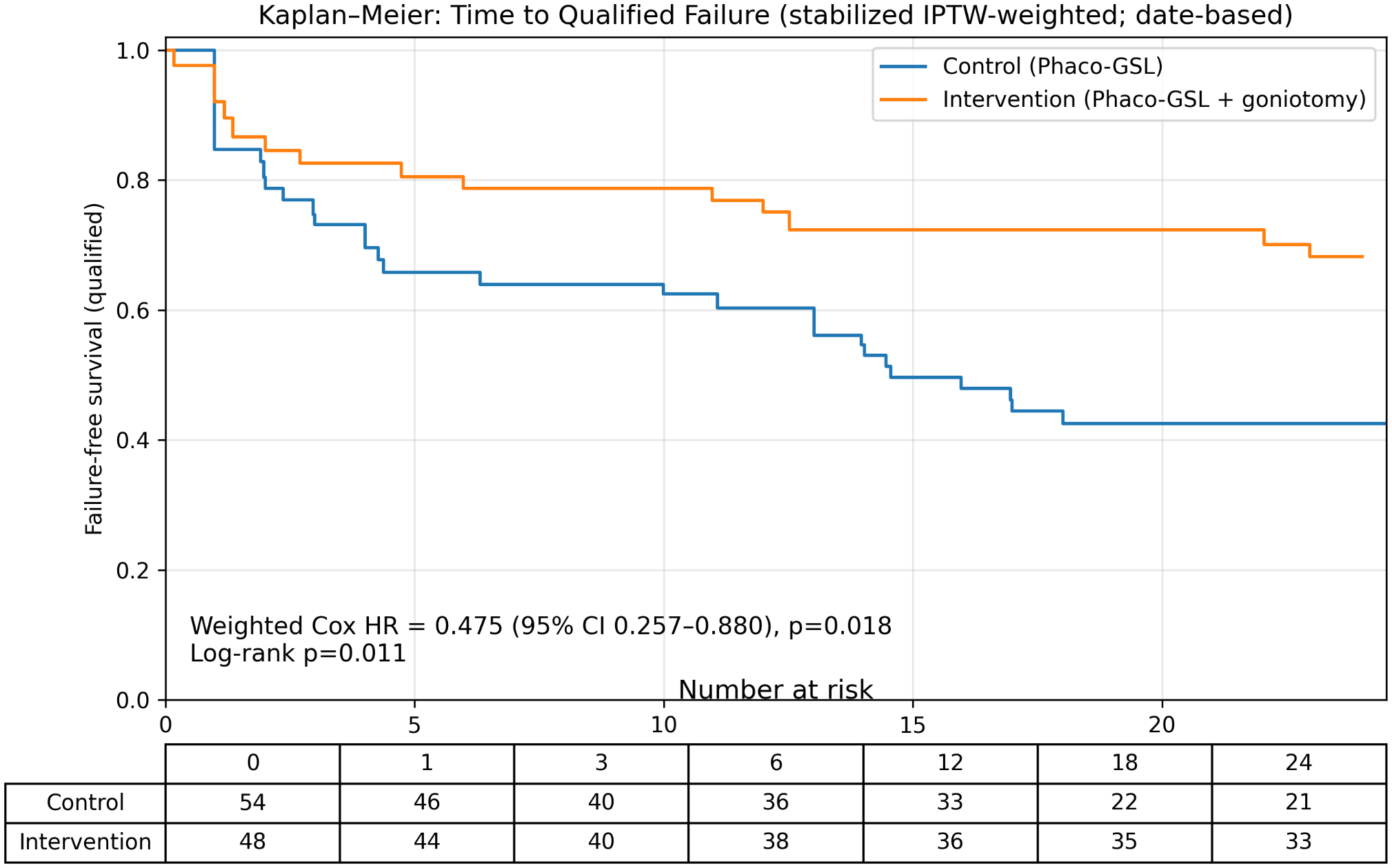


Figure S5. Schematic distinction between date-based and visit-assigned event-time definitions. The schematic is included to clarify definitions and is not a patient-level event-count plot.


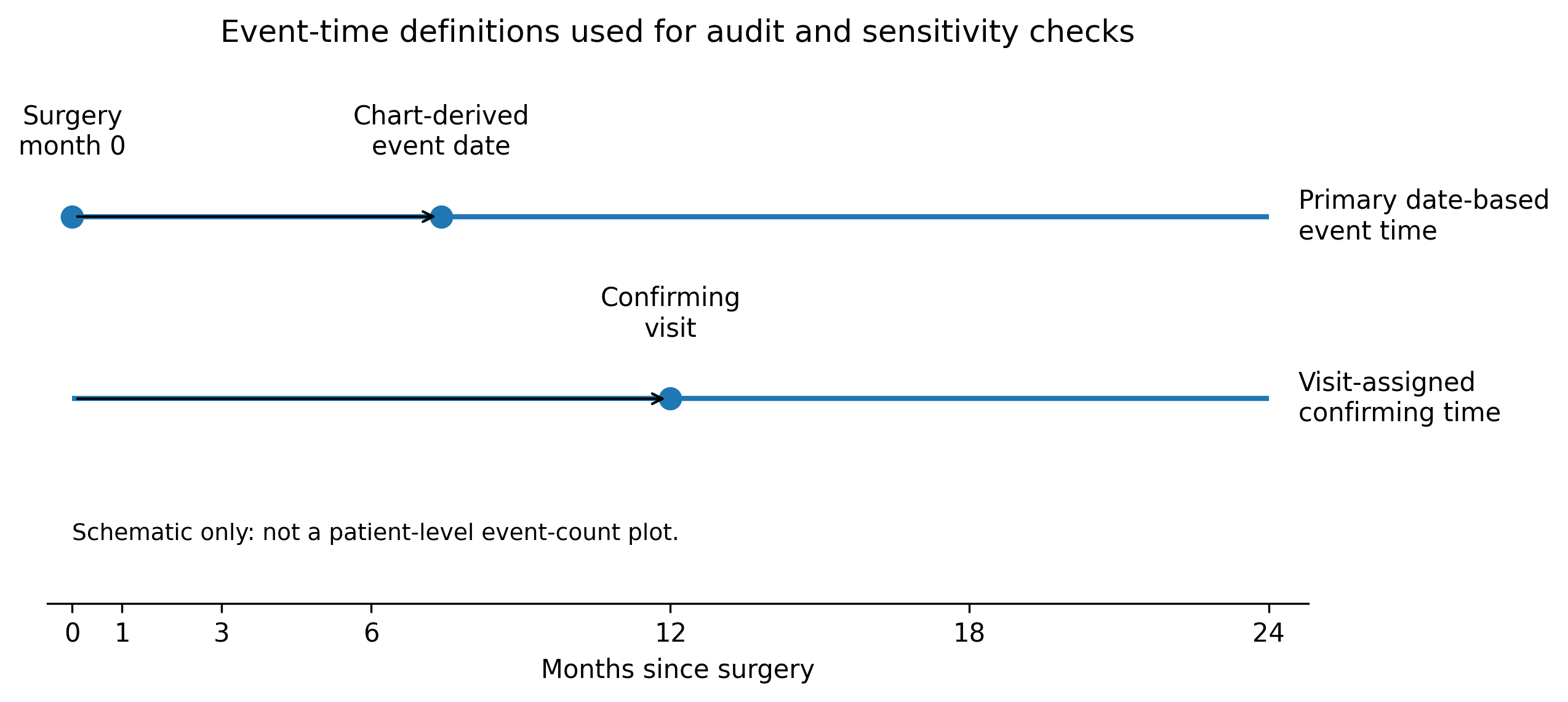


Figure S6. Distribution of cross-fitted predicted individualized benefit estimates (tau-hat) on the risk-difference scale (24-month medication-free complete success).


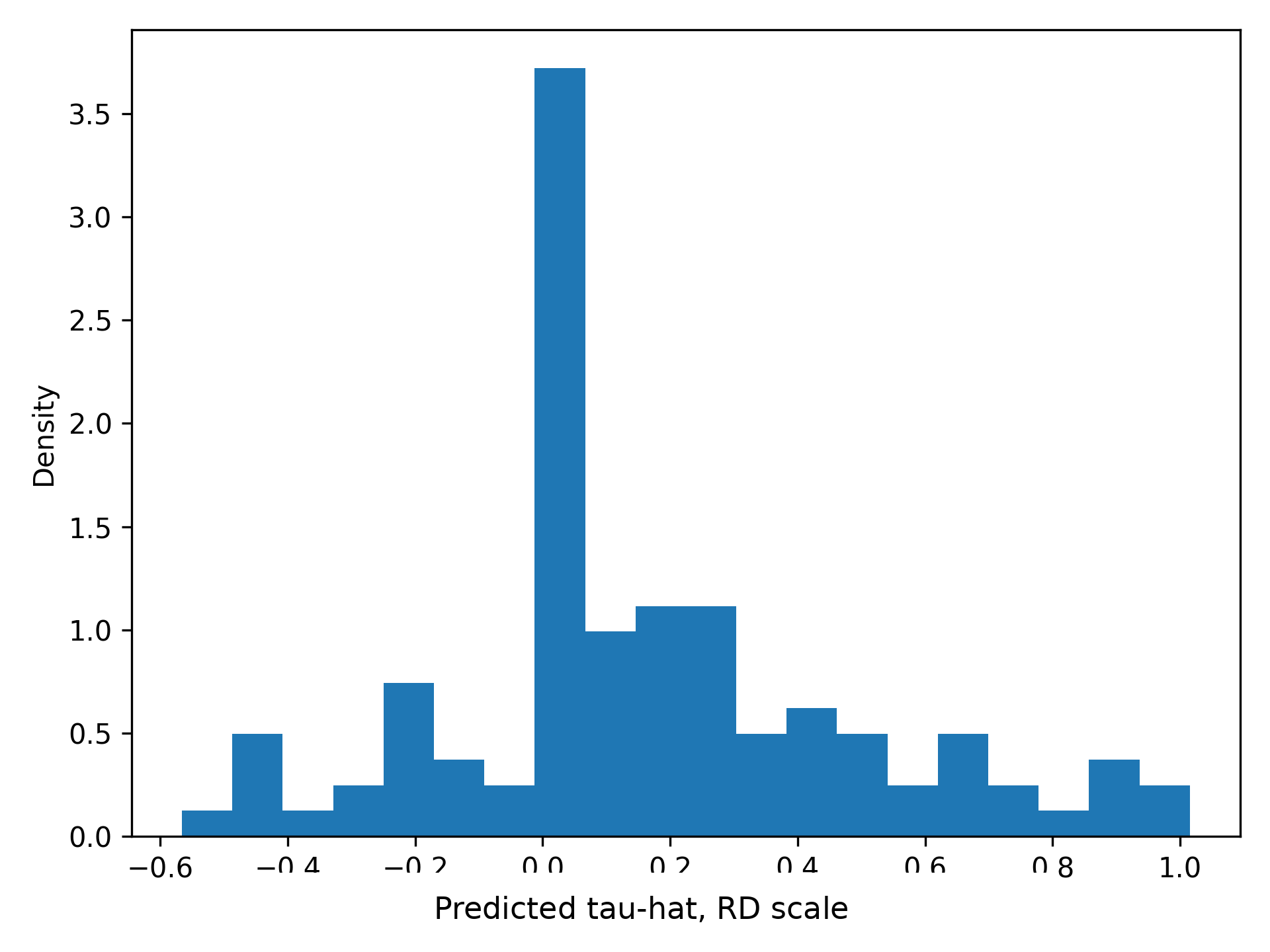


Figure S7. Tau-hat calibration: mean predicted tau-hat vs observed stabilized IPTW risk difference across strata.


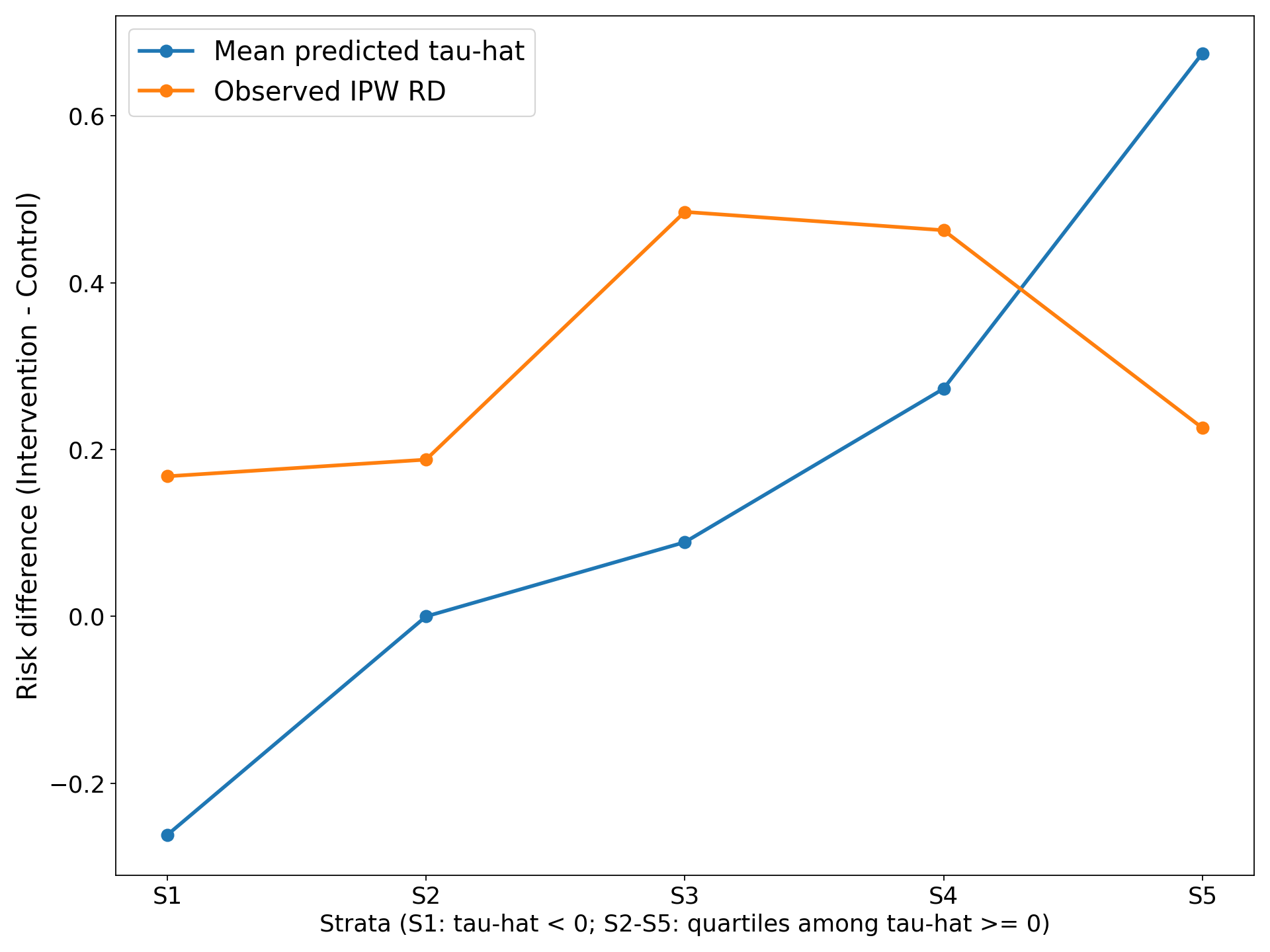


Figure S8. Utility-based net benefit analysis.

Decision-analytic net benefit for 24-month medication-free complete success is plotted against delta, the minimum acceptable absolute benefit on the risk-difference scale. Curves compare candidate strategies, including treat-all, treat-none, phenotype-based rules, and a tau-hat-guided prototype policy (treat if tau-hat >= delta). Higher net benefit at a given delta indicates a preferable strategy under that utility threshold. Results reflect internal proof-of-concept evaluation within the development cohort and should not be interpreted as clinical recommendations.


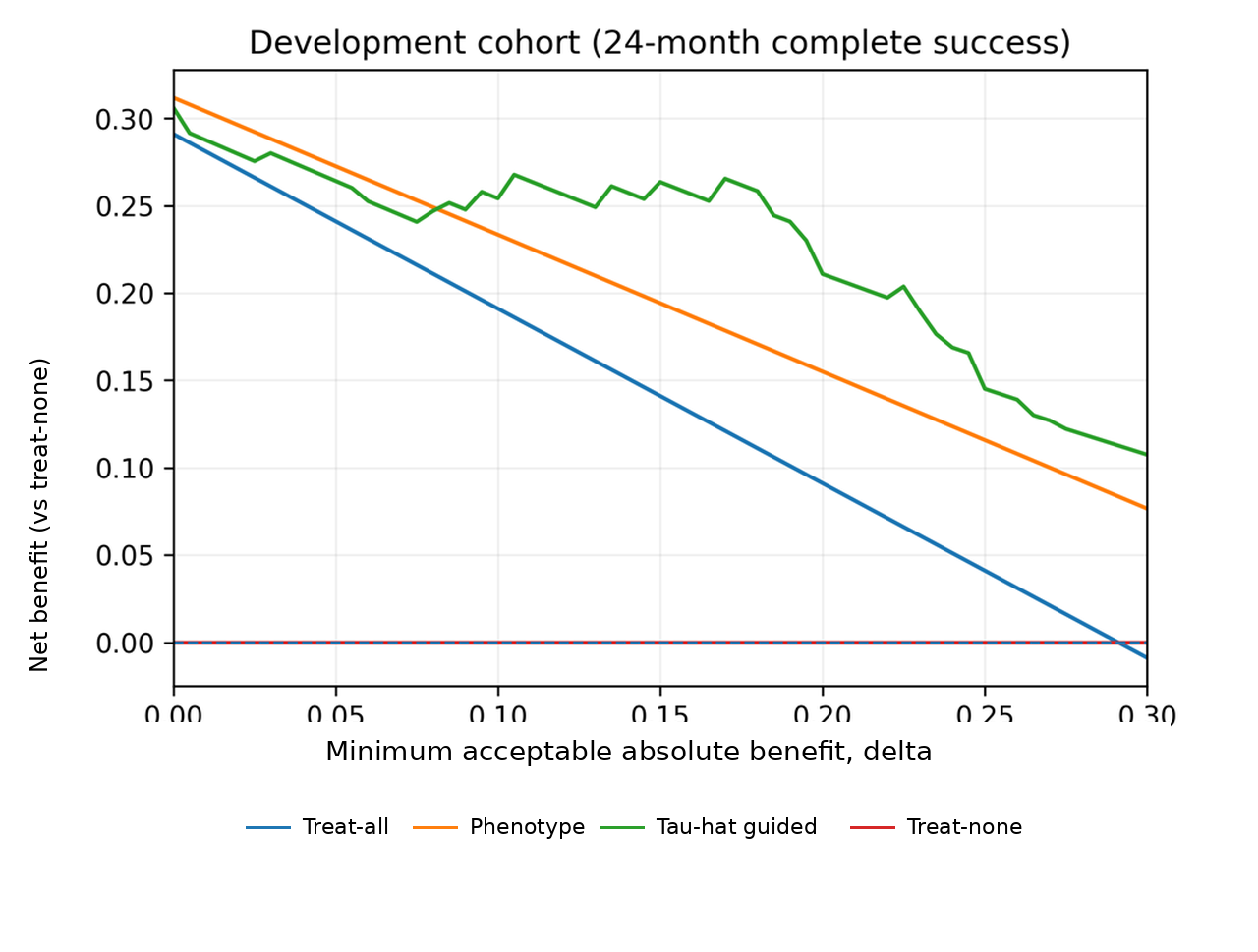

Supplement: Supplementary file 1 [file DataSheet1.docx]
